# Zinc valproic acid complex promotes osteoblast differentiation and exhibits anti-osteoporotic potential

**DOI:** 10.1515/biol-2025-1090

**Published:** 2025-05-12

**Authors:** Huan Wang, Yan Xu, Pan Li, Lingdi Wu

**Affiliations:** Department of Orthopaedics, Lequn Branch, The First Hospital of Jilin University, Changchun, Jilin, 130000, China; Department of Orthopaedics, The 940th Hospital of Joint Logistics Support force of Chinese People’s Liberation Army, Lanzhou, Gansu, 730070, China; Department of Joint Motion Spine, Shaoxing Central Hospital of Zhejiang Province, Shaoxing, Zhejiang, 312030, China

**Keywords:** zinc, valproic acid, osteoporosis, rat osteoporosis model, zebrafish osteoporosis model

## Abstract

This study explores the potential of zinc valproic acid (Z-VA) complex as a promoter of osteoblast differentiation and a preventive agent against osteoporosis. The concentration of 25 µM Z-VA improved osteoblast differentiation by increasing the expression of Runx2 and type 1 collagen mRNA, alkaline phosphatase activity, and cellular calcium deposition. Dexamethasone-induced osteoporosis models were used in zebrafish and rats. In the zebrafish scale regeneration model, Z-VA decreased the hydroxyproline content and tartrate-resistant acid phosphatase activity while also upregulating the calcium to phosphorus molar ratio, Runx2a MASNA isoform, collagen2α, osteocalcin, and osteonectin. Additionally, Z-VA upregulated osteopontin and mitogen-activated protein kinase expression and downregulated matrix metalloproteinase 3 expression. Z-VA increased calcium deposition, callus formation, and bone growth in a zebrafish fracture model. In the rat model, Z-VA increased the bone transverse diameter, length, weight, mineral content, and mineral density, as well as serum Ca^2+^, inorganic phosphate, interleukin-6, tumor necrosis factor alpha, and alkaline phosphatase. Our results suggest that Z-VA may be an effective anti-osteoporotic agent that stimulates bone growth and prevents bone loss. However, further research is needed to elucidate its mechanisms and enhance its therapeutic application.

## Graphical abstract

**Figure:**
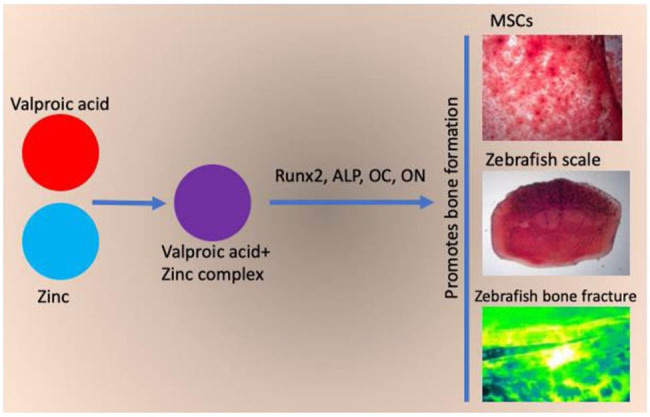

## 1 Introduction

Osteoporosis, a condition affecting bone health, is characterized by decreased bone density and heightened susceptibility to fractures. Globally, approximately 33% of women and 20% of men experience osteoporotic fractures in the hip, spine, and forearm [1]. The condition is divided into two main types: type I, marked by an increased rate of bone turnover, and type II, associated with a reduced rate of bone turnover. Women are more affected by type I osteoporosis due to declining estrogen levels during menopause, which leads to increased bone resorption [2]. In contrast, type II osteoporosis, also known as senile osteoporosis, affects both sexes and results from poor mineralization by osteoblasts and inefficient bone remodeling [1,2]. Bone modeling, involving independent contributions by osteoclasts and osteoblasts, influences growth on the outer bone surface (periosteum). Recent studies suggest that certain medications may impact periosteal bone formation, a factor previously considered unrelated to osteoporosis. Calcium and vitamin D intake are essential for bone health preservation and osteoporosis management. Moreover, engaging in weight-bearing exercises and losing weight, as outlined by Ratajczak et al. [3], may partially reverse osteoporosis. Adherence to vitamin D, calcium supplements, and osteoporosis medications remains low in some patients, and the factors contributing to this poor adherence are investigated in the literature [4].

Pharmacological treatments to reduce fractures aim to increase bone mass using either anti-resorptive or anabolic drugs. Compared to anti-resorptive treatments, fewer anabolic drugs are available. However, long-term adherence to treatment is a significant challenge; only 40% of the patients continue bisphosphonate therapy beyond the first year [5]. Side effects, such as atypical fractures and jaw osteonecrosis, have led to a 50% decrease in bisphosphonate consumption [5]. Valproic acid (VA), a widely used antiepileptic drug, inhibits the CYP450 enzyme with minimal effects on hepatic metabolic enzymes. VA also acts as a histone deacetylase inhibitor, binding to catalytic centers and blocking their activity [6].

VA may cause faulty tubular function in the kidneys, leading to abnormal metabolism and depletion of calcium and phosphorus [7]. While some studies report no adverse effects on bone mineral density (BMD) [8,9], cellular and animal studies suggest that VA may be beneficial for bone health [10,11,12]. However, clinical studies have shown that VA treatment can reduce the BMD [13,14]. This indicates potential strategies for enhancing the positive effects of zinc in combination with VA on bone regeneration, based on previous animal research. Metal ions play critical roles in biological processes, acting as essential cofactors and performing cellular tasks beyond the capability of organic molecules. These ions are often incorporated into drugs for therapeutic purposes [15]. The selection of metal ions with varied oxidation states and coordination environments is a crucial step in developing new metallodrugs for osteoporosis therapy [15]. This study aimed to investigate the anti-osteoporotic potential of zinc valproic acid complex (Z-VA) using dexamethasone-induced osteoporosis models in zebrafish and rats, providing comprehensive insights into its therapeutic potential.

## 2 Materials and methods

### 2.1 Synthesis of Z-VA

The synthesis of zinc(ii) complexes was carried out at room temperature under normal laboratory conditions [16,17]. An aqueous solution of sodium valproate was gradually introduced into a stirred aqueous solution of zinc chloride (Sigma-Aldrich, USA) in a 2:1 molar ratio, resulting in the immediate formation of a white solid. The solid was separated by filtration, washed with cold water, and left to dry naturally. The compound demonstrated solubility in a range of solvents, including methanol, ethanol, acetone, chloroform, dichloromethane, diethyl ether, and ethyl acetate (Sigma-Aldrich, USA). Next, 2,9-dimethylphenanthroline (2,9-Dmphen) (Sigma-Aldrich, USA) (0.92 g, 4.4 mmol) was dissolved in methanol and gradually mixed into a stirred methanol solution of the Z-VA (1.55 g, 2.2 mmol). The mixture was stirred for several hours, after which it was evaporated to leave a solid residue. The resulting solid was collected, washed with ether, and left to air dry.

### 2.2 Cell culture and maintenance

Mouse mesenchymal stem cells (MSCs) (C3H10T1/2) were maintained in Dulbecco’s modified Eagle’s medium (Thermo Fisher Scientific Inc., USA) enriched with 24 mM sodium bicarbonate, 25 mM HEPES, and antibiotics, including penicillin (100 units/mL), streptomycin (100 µg/mL), gentamicin (30 µg/mL), and amphotericin B (2.5 µg/mL). The medium also contained 10% fetal bovine serum sourced from Invitrogen, USA. When the culture reached 70–80% confluence, the cells were passaged using 0.05% trypsin and 0.025 M ethylenediaminetetraacetic acid (Thermo Fisher Scientific Inc., USA).

### 2.3 (3-[4,5-Dimethylthiazol-2-yl]-2,5-diphenyl tetrazolium bromide) (MTT) assay

Cell viability of mouse MSCs was assessed using the MTT assay [18]. Cells were plated at a density of 5,000 cells per well in a 96-well plate and allowed to adhere overnight. The next day, the cells were treated with different concentrations of Z-VA ranging from 0 to 1 mg/mL for 24 h. After the incubation period, 20 µL of 5 mg/mL MTT (Sigma-Aldrich, USA) solution was added to each well and incubated at 37°C for 4 h. After incubation, the medium was removed, and 200 µL of dimethyl sulfoxide (Thermo Fisher Scientific Inc., USA) was added to each well to dissolve the formazan crystals. Absorbance was measured at 570 nm using a microplate reader. Cell viability was calculated relative to untreated control wells to determine the effect of Z-VA on cell viability percentages.

### 2.4 Alizarin red S staining

The experiment began by exposing the cells to different concentrations of Z-VA in a petri dish, and then they were incubated at 37°C in CO_2_ incubators. The culture medium was next removed followed by fixing the cells for 20 min in 95% ethanol. After fixation, the cells were extensively washed with deionized water and stained in a pH range of 4.0–4.5 using 2% Alizarin red S solution (Sigma-Aldrich, USA) for 10 min. After staining, the cells were washed off excess dye and observed under a microscope. To evaluate mineralization, the treated cells were incubated with a solution containing 10% acetic acid and 10% ammonium hydroxide (Sigma-Aldrich, USA). The absorbance of the resulting solution was subsequently measured at 405 nm using a spectrophotometer.

### 2.5 Alkaline phosphatase (ALP) assay

ALP activity of the cells was studied following the ALP assay [19]. The initial protein lysate was prepared using cell lysis with a protein lysis buffer. The lysis buffer used for the ALP activity assay was 1% Triton X-100 prepared in a Tris–HCl buffer (pH 7.5) (Sigma-Aldrich, USA). After that, the resulting cell lysate was incubated with *p*-nitrophenylphosphate (pNPP; Sigma-Aldrich, USA) at 37°C for 30 min. Then, the absorbance of the reaction mixtures was measured at a wavelength of 405 nm using a spectrophotometer for quantification of ALP activity.

### 2.6 Reverse transcription polymerase chain reaction (RT-PCR) study

This study used RT-PCR analysis to examine gene expression patterns of Runx2, type I collagen, alkaline phosphatase (ALP), osteocalcin, and osteonectin in MSCs. The cells were made into osteoblasts after being treated with Z-VA. Subsequently, total RNA was collected from their cells for gene expression investigation. For RNA extraction, the Qiagen RNeasy Mini Kit, USA, was used. Regenerated scales provided a source of differentiated osteoblasts whose gene expression levels were determined by the use of RT-PCR, specifically focusing on certain osteogenic markers such as Runx2a MASNA isoform, type I collagen2α, ALP, osteocalcin, and osteonectin. RNA extraction was done, and its quality was tested using a Nanodrop (Thermo Fisher Scientific, USA), then standardized. Amplification was conducted using a KAPA SYBR FAST qPCR Kit with primer sequences found in supplemental Table S1. The alterations in mRNA folding were assessed using the ΔΔCt technique to precisely measure the levels of gene expression.

### 2.7 Zebrafish maintenance

The zebrafish breeding and maintenance procedure commenced with the segregation of male and female zebrafish into designated tanks for a duration of 2 weeks, where they received live feed twice daily. All research activities were conducted in accordance with the ethical guidelines approved by the institutional animal ethics committee of our institution. After reaching adulthood, zebrafish were provided with commercially available dried worms twice daily as part of their feeding regimen. Tanks were consistently supplied with fresh water and oxygen to uphold a breeding temperature of 27–28°C and maintain a pH range of 5–8. Adult male and female zebrafish were housed separately to facilitate regular breeding cycles, ensuring the continuity of the population.


**Ethical approval:** The research related to animal use complied with all the relevant national regulations and institutional policies for the care and use of animals.

### 2.8 Scale regeneration study

The investigation on scale regeneration began by injecting ten adult zebrafish with a concentration of 10 µM dexamethasone (Sigma-Aldrich, USA) [19]. This was done to establish an osteoporosis model. Subsequently, the fish affected by osteoporosis were treated with Z-VA in order to investigate the process of new bone development, with specific emphasis on the regeneration of scales. Fish that were treated with dexamethasone after administering the osteogenic chemical Z-VA underwent a systematic observation to examine changes in their behavior and physical structure.

The procedure began by killing fish scales with 5% formalin (Sigma-Aldrich, USA) solution on day 14 after anesthesia. The mineralization assessment was conducted on regenerated scales using Alizarin red S and von Kossa staining. For the Alizarin red S staining procedure, fixed scales were immersed in 1% Alizarin red S red solution for 15 min to visualize calcium deposits and subsequently imaged using a fluorescence microscope (Leica Microsystems, Germany). For von Kossa staining, reconstructed scales were incubated in a 5% silver nitrate solution and exposed to ultraviolet light for approximately 15 min to facilitate calcium phosphate visualization. Afterward, excessive stain was removed through rinsing, followed by a treatment with 5% sodium thiosulfate for 5 min to enhance the contrast. The scales underwent subsequent cleaning procedures to ensure clarity and precision in imaging and analysis. A light microscope was used to obtain photo-micrographs of the newly developed scales in order to study osteoblast mineralization. Calcification in the scales was quantified using ImageJ software.

### 2.9 Ca/P ratio by inductively coupled plasma-mass spectrometry (ICP-MS)

ICP-MS was performed to carry out the Ca/P ratio study of the regenerated scales on day 6, as described previously [19]. The scales were first removed neatly and then treated with 61% nitric acid (Sigma-Aldrich, USA) (100 µL) in a hot plate adjusted to 230°C for 5 h. After that, a 50 µL sample mix was extracted and diluted by adding 1% dilute nitric acid by volume in which it was stored at a temperature of 4°C. The ICP-MS method was used to determine the Ca/P molar ratio, which provided accurate quantification of Ca and P mineral contents in scales, allowing for precise investigation of mineralization kinetics.

### 2.10 Tartrate-resistant acid phosphatase (TRAP) and hydroxyproline activity

For TRAP activity, we adopted the following approach using reconstructed zebrafish scales: scales were first put into a solution containing 0.1 M sodium acetate buffer supplemented with 20 mM tartrate for an hour. Then, additional incubation was carried out with a solution made up of 20 mM tartrate, 0.1 M sodium acetate buffer, and pNPP (Sigma-Aldrich, USA). After addition of concentrated NaOH, the enzymatic reaction was stopped followed by measurement of absorbance of TRAP activity at a wavelength *λ* of 405 nm where absorption maximum occurs. For hydroxyproline content estimation, reconstructed scales from the fish underwent thorough washing with distilled water followed by drying. For this assay, the Sigma-Aldrich Hydroxyproline Assay Kit was employed. The dried samples were then placed in a silica crucible and subjected to calcination at 800°C to obtain cracked powder. The weight of the resulting powder was measured, and subsequently it underwent hydrolysis using 6 M hydrochloric acid for detection with a hydroxyproline kit (Thermo Fisher Scientific Inc., USA).

### 2.11 Osteoporosis rat model

In order to create an osteoporosis rat model, as described in the study of Luo et al. [20], female Wistar rats weighing between 180 and 200 g were obtained from the Laboratory Animal Centre at the Huzhou Institute for Food and Drug Control. The rats were kept in a controlled environment in a vivarium maintained at a temperature of 25°C, with a relative humidity of 65% and a light–dark cycle of 12 h of light followed by 12 h of darkness. They were provided with unrestricted access to food and drink throughout the entire study period. The animal handling and treatment protocols adhered strictly to the rules specified in the National Institutes of Health Guide for Care and Use of Laboratory Animals. The institution’s ethics committee gave approval for conducting the research. Sixty Wistar rats were placed in one of the four groups of control, dexamethasone, alendronate, and Z-VA (10, 20, or 40 mg/kg, respectively). After a fasting period of 12 h, the control group animals received normal saline, while those in the other groups were given oral dosages of either 1 mg/kg alendronate or 10, 20, or 40 mg/kg Z-VA, respectively, once per day for 8 weeks. Similarly, in order to induce osteoporosis, these groups’ rats were administered with intramuscular injections of dexamethasone, twice per week for 8 weeks; controls received oral doses of normal saline and intramuscular injection of normal saline following the same regimen. The necessary concentrations were obtained by diluting alendronate or Z-VA with normal saline, so that each rat received an intragastric volume of 20 mL/kg. Weekly body weight measurements were made on all rats for the entire duration of 8 weeks using an electronic balance. On day 57, the animals were deeply anesthetized with 10% chloral hydrate (Sigma-Aldrich, USA) (3.0 mL/kg) administered via the intraperitoneal injection route. Then, abdominal aortic blood was collected and spun in a centrifuge to obtain serum followed by refrigeration at −20°C for use in future studies. Furthermore, left and right femurs of each rat were immediately separated, rinsed with normal saline to remove any residual blood, and then snap-frozen at −80°C for more analysis. Measurements of bone tissue parameters on the right femur, such as the length, transverse diameter, weight, BMC (bone mineral content), and BMD, were determined. Length and transverse diameter measurements were obtained using a Vernier caliper, whereas weight was measured with an electronic balance. A BH41-HH6005 single-photon BMD meter was used for measuring the BMC and BMD.

### 2.12 Enzyme-linked immunosorbent assay (ELISA) protocol

Serum biochemical indices, including Ca^2+^, inorganic phosphate (IP), interleukin-6 (IL-6), tumor necrosis factor-alpha (TNF-α), and ALP, were quantified using commercially available ELISA kits (Thermo Fisher Scientific Inc., USA), following the manufacturers’ instructions. Briefly, serum samples were appropriately diluted and added to pre-coated microplates specific to each analyte. After incubation with the primary antibody, unbound components were removed by washing, followed by the addition of the enzyme-conjugated secondary antibody. The reaction was developed using a chromogenic substrate, and absorbance was measured at the appropriate wavelength using a Thermo Scientific microplate reader (Waltham, MA, USA). The concentrations of the biochemical markers were determined by extrapolating the absorbance values onto standard calibration curves generated for each assay [21].

### 2.13 Statistical analysis

All experiments in this study were carried out three times, so that reliability and repeatability of data could be ensured. The mean values with standard deviation (mean ± SD) showed how well data were distributed within experimental groups and their variability between them clearly. By utilizing Student’s *t*-test, statistical differences between experimental groups demonstrating *p* < 0.05 as the significance threshold became apparent after analysis. For comparing means between two groups, Student’s *t*-test was appropriate, assuming normal distribution and equivalent variation within each group, ensuring robust and accurate statistical analysis. SPSS Statistics, version 20.0 (IBM Corp., Armonk, NY, USA) software was used for statistical analysis.

## 3 Results and discussion

### 3.1 Biocompatible nature of Z-VA in mouse MSCs

**Figure 1:**
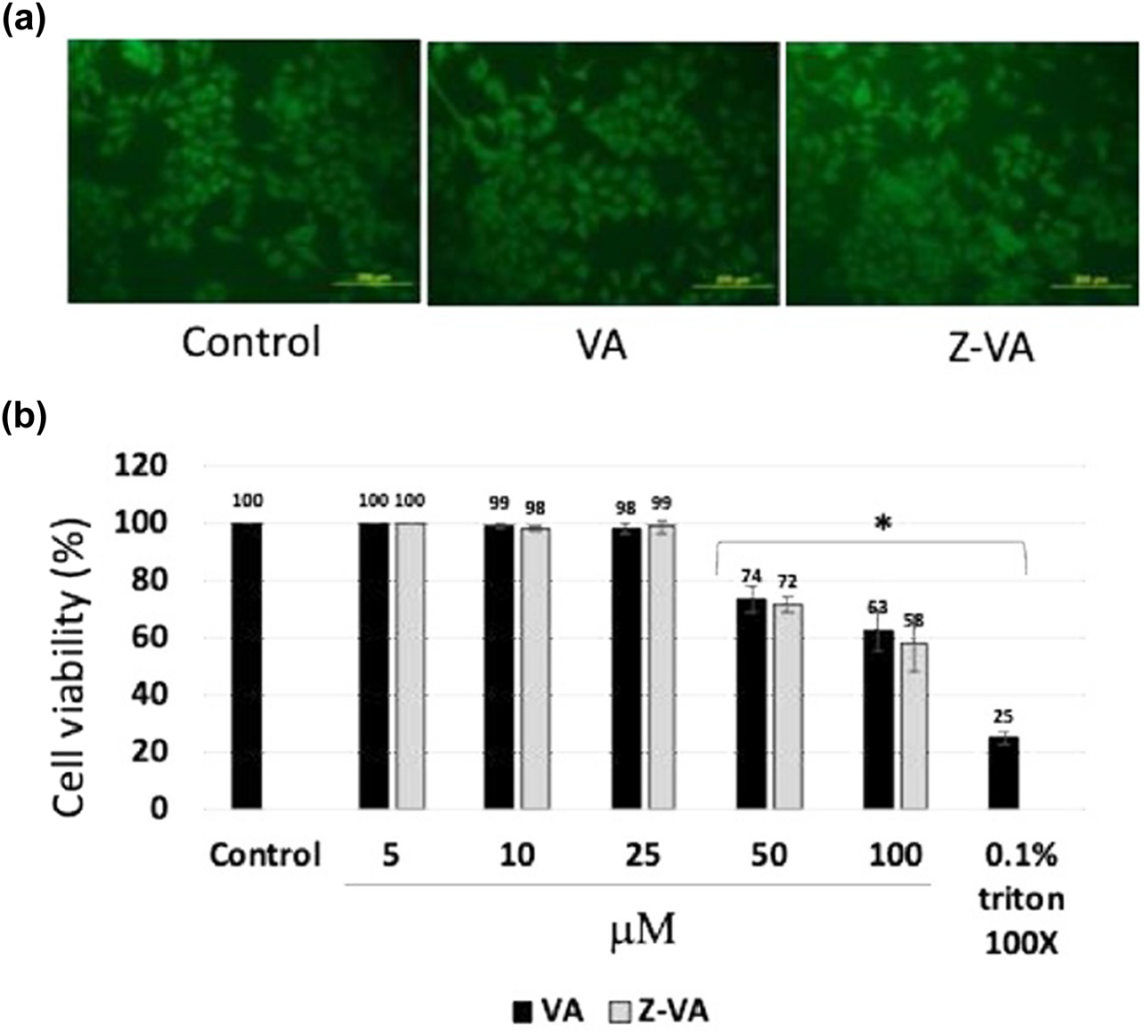
VA and Z-VA biocompatibility assay in mouse MSCs. (a) Photomicrographs illustrating matured osteoblast cells stained with FDA after 1 week of MSC differentiation. (b) Results of the MTT assay depicting the toxicity profile of VA or Z-VA. Scale bar: 200 µm.

Z-VA’s effects on mouse MSCs were studied *in vitro* to determine its efficacy in promoting differentiation. Mouse MSCs initially received different doses of VA or Z-VA (0–100 µM), and the cell viability was assessed by the MTT assay as an indicator for cytotoxicity (Figure 1b). These results showed no noticeable toxic effects with Z-VA treatment or VA. After exposure to either VA or Z-VA (25 µM), fluorescein diacetate (FDA) staining was carried out to observe the morphological changes in the cells (Figure 1a). Therefore, the capacity of MSCs to differentiate into osteoblasts is very important for bone tissue regeneration and repair. Our data demonstrate that there are no toxic effects of Z-VA on mouse MSCs, thus proving its safety for future therapeutic purposes. Moreover, the absence of notable morphological changes in MSCs following Z-VA exposure further supports its biocompatibility. These preliminary results lay the groundwork for further investigation into the osteogenic potential of Z-VA, shedding light on its role in promoting osteoblast differentiation and bone regeneration. Further studies, including gene expression analysis and functional assays, are warranted to elucidate the underlying mechanisms and optimize the Z-VA concentration for enhancing the osteogenic differentiation of MSCs.

### 3.2 Z-VA promotes MSCs toward osteoblast differentiation at the cellular level

**Figure 2:**
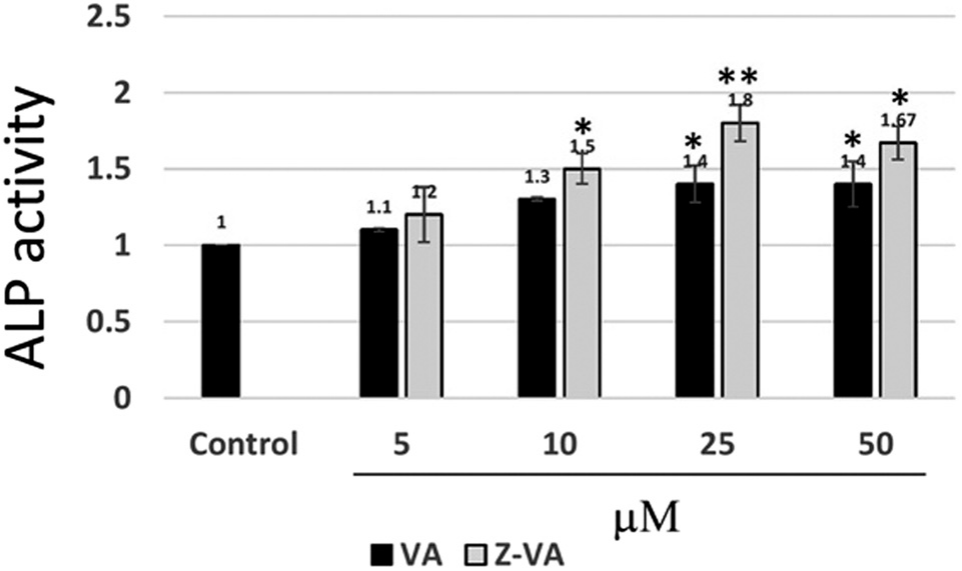
ALP activity. Differentiated stem cells exposed to various concentrations of VA or Z-VA (0–100 µM) demonstrating variable ALP activity during osteoblastic differentiation. The bar diagram depicts the heightened ALP values observed for 0–50 µM Z-VA in differentiated osteoblasts. *Significantly different from control (*p* < 0.05). **Significantly different from VA (*p* < 0.05).

The ALP assay was used to confirm the differentiation of mouse MSCs into osteoblasts, which is essential for bone formation. ALP, which is a tetrameric surface glycoprotein, helps regulate bone matrix arrangement by aligning it with minerals such as calcium and phosphates [22]. In our *in vitro* analysis, we assessed the ALP activity associated with VA or Z-VA during bone remodeling. Mouse MSCs were exposed to VA or Z-VA at varying concentrations (0–50 µM) for 7 days, after which ALP activity was assessed (Figure 2). The results indicated that 25 µM of either VA or Z-VA significantly enhanced the ALP activity compared to other concentrations. This concentration appeared to be the most effective in stimulating ALP activity under the tested conditions. As a significant osteogenic marker, ALP signifies osteoblastic activity in new bone development [23]. Therefore, it can be concluded that Z-VA significantly enhances ALP activity compared to control and VA-treated cells, thus indicating its strong potential to stimulate osteoblast differentiation [24].

**Figure 3:**
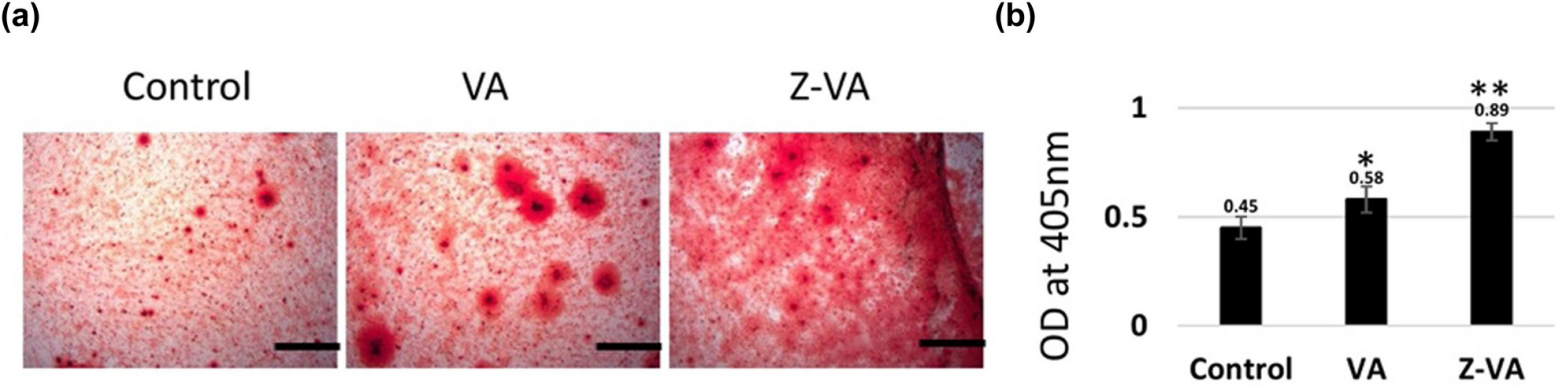
Mineralization assay on stem cell differentiation. (a) Alizarin red S red staining of mouse MSCs (C3H10T1/2) showing calcium mineralization during week 1. Photomicrographs clearly illustrating the formation of mineralized nodules in differentiated osteoblasts. Scale bar: 25 µm. (b) Bar diagram displaying absorbance values (405 nm) for Z-VA on Alizarin red S staining during 7 days compared to untreated control/VA. *Statistically significant compared to control (*p* < 0.05). **Significantly different from VA (*p* < 0.05). The scale bar indicates 200 µm.

Moreover, the extent of calcium deposition on newly differentiated osteoblasts was further confirmed by the Alizarin red S staining assay for mineralized nodule formation. Osteoblasts treated with VA or Z-VA had bright nodules indicating mineralization, as shown in Figure 3a. The absorbance values at 405 nm were quantitatively analyzed to demonstrate that there was an increase in calcium mineralization when stem cells were exposed to Z-VA compared to control and VA-treated cells over 7 days (Figure 3b). The development of mineralized nodules is strongly associated with the deposition of calcium, a process that is greatly enhanced by Z-VA, thereby aiding in the deposition of minerals during the production of new bone [25]. The results emphasize the crucial importance of Z-VA in stimulating the formation of bone cells and improving the process of mineralization in MSCs, indicating its potential as a treatment for osteoporosis. Further investigations into the underlying molecular mechanisms and long-term effects of Z-VA treatment are warranted to elucidate its therapeutic efficacy in bone regeneration and osteoporosis management.

### 3.3 Z-VA enhances the expression of osteoblast marker genes during the differentiation of MSCs into osteoblasts

**Figure 4:**
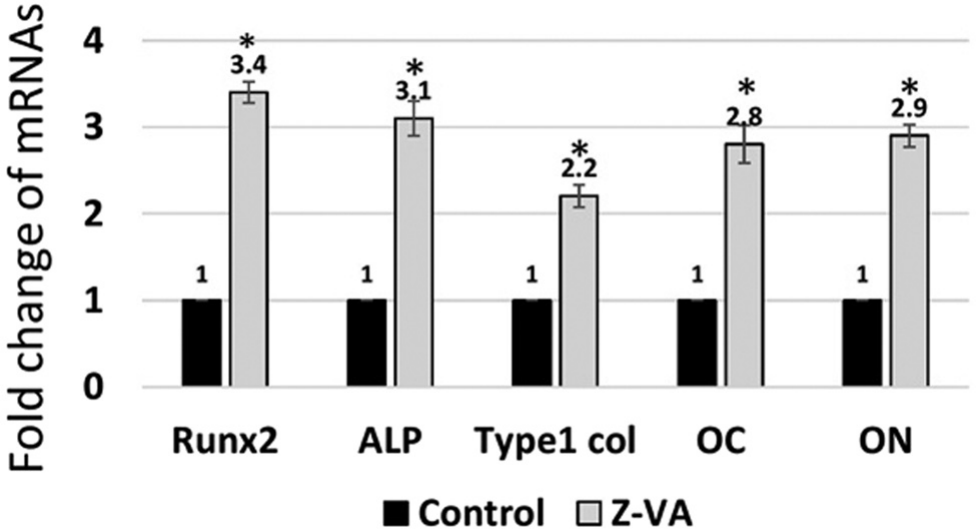
Z-VA promotes osteoblast markers. Expression of Runx2, ALP, type I collagen, osteocalcin, and osteonectin in MSCs during 7 days of Z-VA induction. *indicates a significant increase compared to control (*p* < 0.05).

Research on osteogenic cell signaling markers through the use of RT-PCR analysis provided great insight into MSC differentiation following Z-VA treatment. Differentiated MSCs were tested for key osteogenic markers such as Runx2, type I collagen, ALP, osteocalcin, and osteonectin, as shown in Figure 4. The results indicated that Runx2 and type I collagen expression levels were significantly increased in VA and Z-VA-treated groups compared to the control. This suggests that VA or Z-VA are osteo-inductive, promoting osteogenesis through the upregulation of major bone-forming growth factors [26]. Elevated levels of ALP, osteocalcin, and osteonectin observed after Z-VA treatment highlight its significance in facilitating new bone formation by promoting enhanced mineralization potential [27]. In particular, ALP, a critical marker for differentiation of preosteoblasts, plays an important role in bone remodeling. The high expression level of this enzyme in processed cells hints at the important function it plays during bone repair, which includes but is not limited to cell–matrix interaction as well as collagen binding with bone mineralization [20]. This enhancement of ALP expression adds to the rise of inorganic phosphate concentration, which helps in mineralization and lowers the levels of extracellular pyrophosphate, a mineral formation inhibitor [28]. Similarly, the higher levels of Runx2 and type I collagen signify their activation through the MAPK pathway. In this context, the adhesion of type I collagen to other cell adhesion molecules increases through this activation, thereby enhancing bone matrix formation and development [28,29,30]. Acting as an essential transcription factor for osteoblastic differentiation, Runx2 stimulates the expression of various osteoblast marker genes, including ALP, type I collagen, osteocalcin, and osteonectin, thereby facilitating different aspects related to osteoblast differentiation such as bone matrix formation and mineralization. All these results demonstrate that Z-VA significantly promotes gene expression of specific mRNAs or enzymes related to bone mineralization and plays a role in new bone generation. Its potential to regulate cellular functions at a molecular level and its ability to possibly inhibit mechanisms that contribute to bone resorption underline its promise as a therapeutic agent for the treatment of osteoporosis and promotion of bone repair. Overall, the observed effects of Z-VA on osteoblast differentiation and mineralization represent a promising avenue for addressing osteoporosis-related bone disorders. Further exploration into its underlying mechanisms and long-term effects is imperative to establish its therapeutic efficacy and safety profile in clinical settings.

### 3.4 Impact of Z-VA on calcium deposition in zebrafish scale regeneration

**Figure 5:**
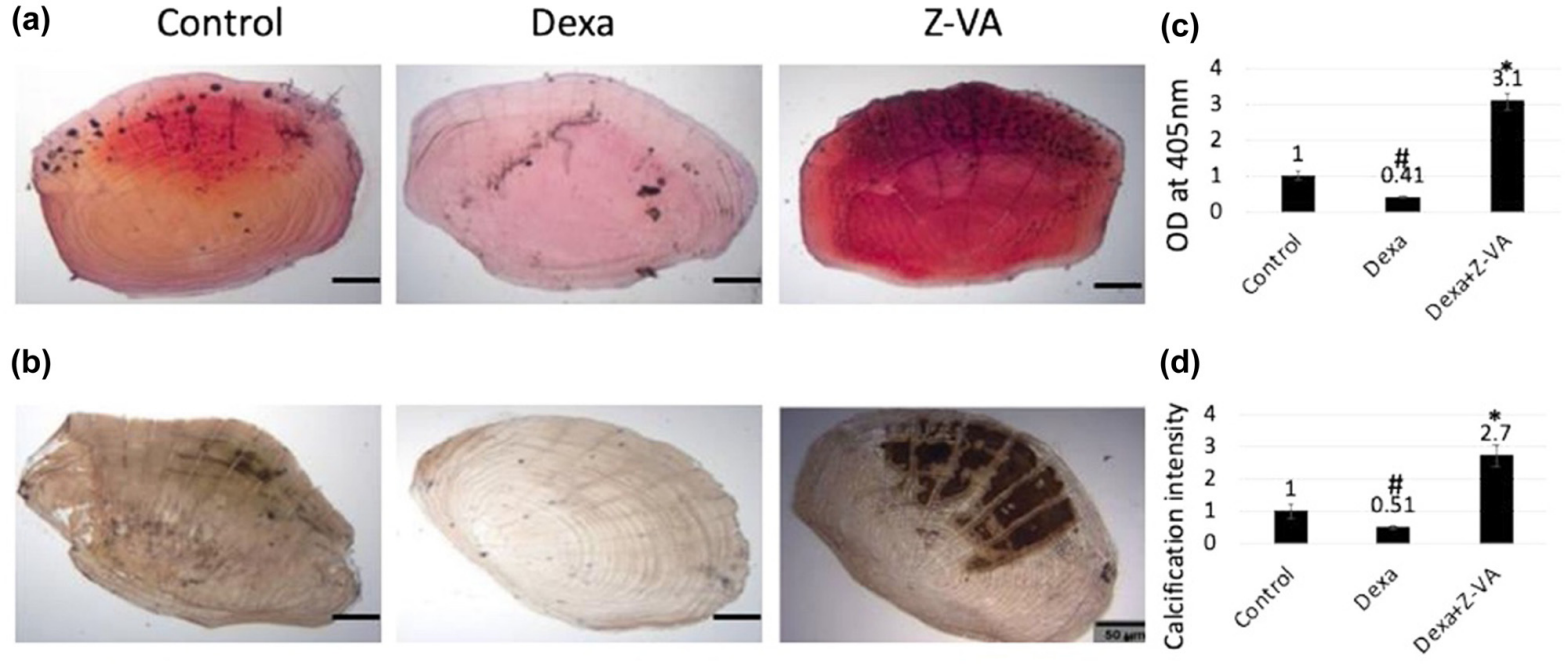
Effect of the anti-osteoporotic agent Z-VA on scale mineralization. (a) Regenerated scales observed from the Alizarin red S red staining assay exhibit red nodules, indicating calcium deposition facilitated by osteoblast migration from the focal region toward the periphery. (b) Photomicrograph from the von Kossa staining assay reveals calcified brown-colored scales with calcium and phosphate mineralization. Non-mineralized regions resulting from osteoclastic matrix degradation are highlighted by white-colored resorption pits. Bar diagram depicting the absorbance of (c) Alizarin red S red and (d) von Kossa stained scales, illustrating calcification during week 4. *indicates a significant increase compared to control/Dexa, while #indicates a significant decrease compared to control (*p* < 0.05). The scale bar indicates 50 µm.

In our study, we established a dexamethasone-induced zebrafish osteoporosis model to evaluate the effects of Z-VA on scale regeneration using Alizarin red S red and von Kossa staining assays. Our experimental findings demonstrated significant effects on the regenerated scales in Z-VA-treated fish, notably observed in increased calcium deposition compared to both the control and dexamethasone-induced groups by week 3, as depicted in Figure 5a. This augmentation in calcium deposition suggests the induction of bone-related factors by the anti-osteoporotic compound, resulting in the development of well-formed scales even in dexamethasone-induced fish [31].

Moreover, the cracked bone surfaces affected by osteoclastic demineralization, induced by dexamethasone, were effectively replaced with bone-forming osteoblasts, as evidenced by the well-defined circular index at the regenerated site (Figure 5b: von Kossa staining). In contrast, the groups treated with dexamethasone showed a decrease in calcium deposition and a reduction in bone density in the distorted areas, suggesting impaired bone regeneration [32]. Quantitative analysis through bar diagrams further supported the enhanced bone mineralization capacity of scales in response to Z-VA treatment, which was observed with Alizarin red S red staining at an absorbance of 405 nm (Figure 5c). Similarly, the Z-VA-treated groups showed a higher intensity of calcification compared to the control and dexamethasone groups when evaluated using von Kossa staining (Figure 5d). These results suggest that Z-VA, in conjunction with dexamethasone, facilitated pronounced osteoblastic relocation from the central to the frontal scale region, thereby facilitating calcium deposition that is crucial for the generation of new scales [19]. Overall, our findings underscore the potential of Z-VA as an effective therapeutic agent for mitigating osteoporosis and promoting bone regeneration in zebrafish scale models. However, further investigation into its underlying mechanisms and long-term effects is essential to fully elucidate its therapeutic efficacy and safety profile in the context of bone disorders.

### 3.5 Z-VA decreases TRAP and hydroxyproline activity

**Figure 6:**
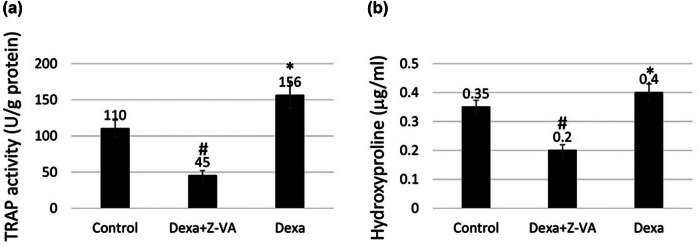
TRAP and hydroxyproline activity. Illustration of the impact of TRAP (a) and hydroxyproline content (b) in the regenerated scales upon treatment with anti-osteoporotic Z-VA in an osteoporosis-induced zebrafish model. *indicates a significant increase compared to control/Dexa, while #indicates a significant decrease compared to control (*p* < 0.05).

The balance between osteoclastic and osteoblastic activity is crucial for the mineralization process of the bone matrix. Osteoclastic activity, characterized by the presence of resorption lacunae in the scales, can impede osteoblastic activity, leading to a reduction in mineral deposition [33]. Hence, we investigated the impact of the key marker TRAP on bone resorption in regenerated scales. As illustrated in Figure 6a, the percentage of TRAP activity was assessed in control, dexamethasone-treated, and dexamethasone-Z-VA-treated fish with regenerated scales.

Quantitative analysis of the TRAP assay revealed a significant reduction in enzymatic activity in scales treated with dexamethasone-Z-VA compared to both control and dexamethasone-treated groups. TRAP, a critical marker of osteoclastic activity, catalyzes hydrolytic reactions within the resorption lacunae of scales [28]. Treatment with anti-osteoporotic Z-VA decreased the localization of osteoclasts within the resorption lacunae, particularly in the grooves, while osteoblasts occupied the posterior margin [34]. Osteoclasts recruited from the monocyte lineage are responsible for mineral resorption in the episquamous calcified layer, facilitating bone matrix formation [35]. These findings confirm that TRAP regulates osteoclastic activity for bone resorption and new bone formation.

Hydroxyproline is a bone biomarker associated with bone resorption [32]. It belongs to the group of bone collagens that are broken apart when bones disintegrate. Therefore, understanding the hydroxyproline activity during the bone remodeling process becomes crucial [32]. The experimental results showed that there was a significant decrease in the release of hydroxyproline in the dexamethasone-Z-VA-treated group during scale regeneration compared with the control and dexamethasone-induced groups (Figure 6b). The rise in levels of hydroxyproline indicates that osteoclasts play an important role in scale resorption and remodeling during zebrafish scale repair induced by osteoporosis. Scale renewal relies on enzymatic functioning in forming cells synthesizing scales for reabsorption. Moreover, growth factors and matrix proteins such as collagen and its degradation products released by hydroxyproline are required for this regenerative process [36]. The elevated levels of hydroxyproline observed in recently regenerated scales confirm the phase of scale healing involving degradation of the existing scale bone matrix, resulting in the production of new scale bone. In conclusion, our findings underscore the potential of Z-VA in reducing osteoclastic activity and promoting bone regeneration in the zebrafish scale model. This emphasizes the importance of matrix proteolysis in regulating bone resorption and remodeling during new scale formation. Further exploration of these mechanisms is imperative for understanding Z-VA’s therapeutic efficacy in bone disorders.

### 3.6 Z-VA promotes Ca/P ratio in zebrafish regenerated scales

**Figure 7:**
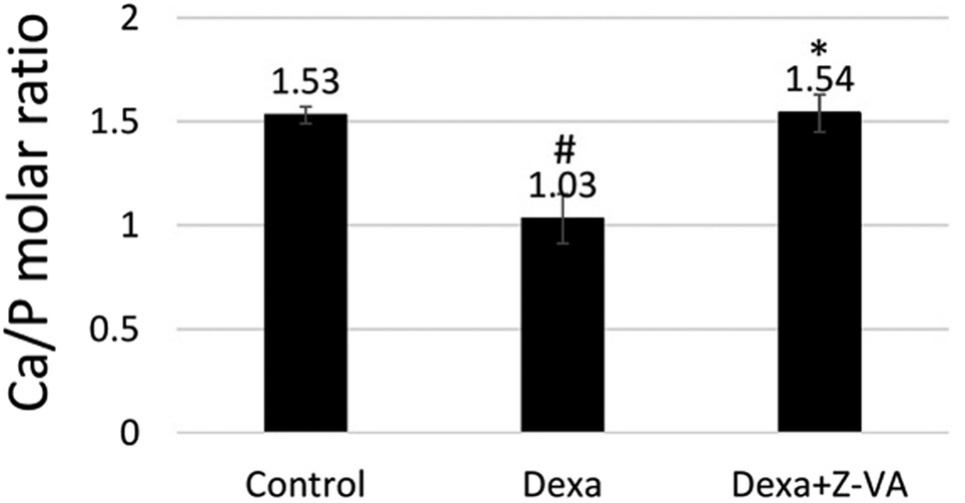
Impact of Z-VA on bone minerals. After treatment, the regenerated scales were analyzed for Ca/P ratio by ICP-MS analysis. *indicates a significant increase compared to control/Dexa, while #indicates a significant decrease compared to control (*p* < 0.05).

The impact of dexamethasone on regenerated scales was evaluated by quantifying their calcium and phosphorus contents [37]. Figure 7 illustrates the calcium-to-phosphorus (Ca/P) ratio of the regenerated scales on day 6 post-osteoporosis induction. Analysis revealed that fish treated with dexamethasone-Z-VA displayed higher Ca/P ratio values compared to both control and dexamethasone-treated groups on day 6 of regeneration. However, previous studies have suggested that the calcium-to-phosphorus ratio typically hovers around 1.55 for hydroxyapatite in native bone [38]. Hence, the observed increase in the Ca/P ratio of the regenerated scales suggests a potential decline in osteoclastic activity, with no significant alteration in their crystalline phase [39]. These findings suggest that Z-VA treatment might enhance mineralization and preserve the bone matrix composition, underscoring its potential therapeutic effectiveness in bolstering bone health and regeneration. Further investigations are warranted to unveil the mechanisms underlying these effects and to evaluate the long-term implications of Z-VA treatment on bone tissue structure and function.

### 3.7 Z-VA promotes osteoblast markers in regenerated scales

**Figure 8:**
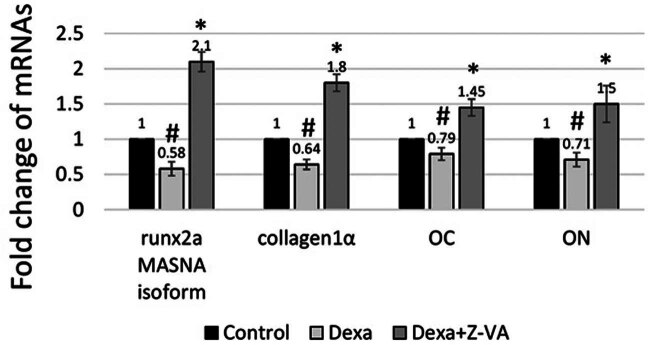
Analysis of bone markers. Treatment with dexamethasone–Z-VA resulted in a significant alteration in Runx2a MASNA isoform, collagen2α, osteocalcin, and osteonectin mRNA expression. *indicates a significant increase compared to control/Dexa, while #indicates a significant decrease compared to control (*p* < 0.05).

Gene expression analyses of mRNA were performed on the regenerated scales of zebrafish with induced osteoporosis to investigate the bone resorptive and bone-forming characteristics of Z-VA. The expression levels of important indicators, such as Runx2a MASNA isoform, collagen2α, osteocalcin, and osteonectin, were assessed by RT-PCR analysis. Figure 8 illustrates the evaluation of osteogenic markers in the regenerated scales after treatment with control, dexamethasone, and dexamethasone-Z-VA groups. The findings showed a substantial increase in the levels of Runx2a MASNA isoform and collagen2α in the dexamethasone-Z-VA group, compared to both the control group and the dexamethasone-treated group. In contrast, the groups treated with dexamethasone exhibited decreased levels of osteogenic markers, suggesting the presence of osteoporosis. The upregulation of these bone markers in the dexamethasone-Z-VA group suggests that the anti-osteoporotic Z-VA has the ability to promote bone healing. This is achieved by modulating osteogenic cellular signaling through osteoblast activity and facilitating collagen production during the process of bone remodeling.

Osteoblastic differentiation, maturation, and proliferation are pivotal in maintaining bone metabolism by regulating cell signaling mechanisms associated with various signaling messengers and markers. Enhanced expression of bone-forming markers like Runx2a MASNA isoform and collagen2α can trigger other osteogenic genes such as osteonectin and osteocalcin on the cell membrane, promoting bone matrix maturation and mineralization [19]. Osteonectin and osteocalcin, as extracellular matrix proteins, contribute significantly to osteoblastic differentiation and mineralization guided by osteoblasts.

Additional experimental findings demonstrated that Z-VA increased the expression of both osteocalcin and osteonectin in the regenerated scales, compared to the control and dexamethasone-treated scales. Osteonectin interacts with hydroxyapatite, collagen, and other molecules involved in cell adhesion, promoting the development of the bone matrix. Osteocalcin has the ability to bind to calcium and phosphorus minerals, which in turn stimulates the development of apatite, stabilizes collagen, and promotes mineralization in the freshly formed bone.

Overall, the upregulation of Runx2a MASNA isoform and collagen2α indicates that Z-VA, an anti-osteoporotic compound, may have a positive effect on osteoblastic differentiation. This, in turn, can enhance the expression of osteonectin and osteocalcin, leading to the maturation and mineralization of the bone matrix, ultimately promoting the regeneration of new bones.

### 3.8 Regulation of Z-VA’s in matrix metalloproteinase 3 (MMP3)–osteopontin (OPN)–MAPK signaling

The complex mechanisms that regulate bone metabolism, including the development, maturation, growth, and death of bone-forming cells, depend greatly on transmembrane signaling channels that coordinate cellular reactions and bone healing processes [28]. Among these pathways, the mitogen-activated protein kinase (MAPK) pathway is an important controller of signals from outside the cell, coordinating the cellular responses involved in bone formation at the molecular level. This pathway involves a series of active protein kinases that carefully control the transmission of signals [28].

**Figure 9:**
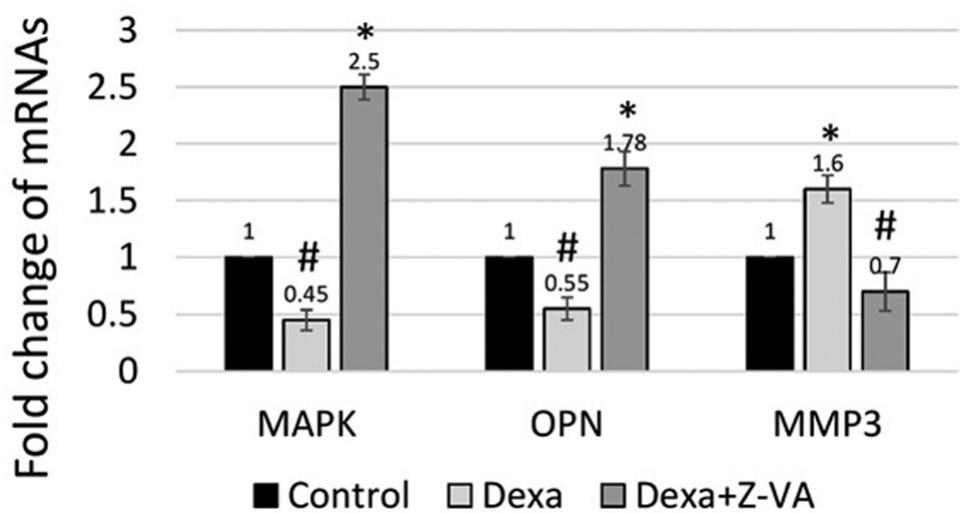
Z-VA plays a regulatory role in MMP3–OPN–MAPK signaling. Real-time RT-PCR results demonstrating the expression of MAPK, osteopontin, and MMP3 mRNA levels. *indicates a significant increase compared to control/Dexa, while #indicates a significant decrease compared to control (*p* < 0.05).

Figure 9 illustrates that zebrafish treated with Z-VA showed significantly higher levels of MAPK and OPN compared to both the control and dexamethasone groups (Figure 9). The results suggest that administering Z-VA during the regrowth of scales significantly increases the protein concentrations of OPN and MAPK. In contrast, administration of dexamethasone resulted in reduced levels of OPN and MAPK, together with elevated levels of MMP3, in zebrafish larvae with induced osteoporosis.

A recent study highlights the strong correlation between MAPK and osteoblastic differentiation, maturation, proliferation, and apoptosis [36]. Crucially, studies have demonstrated that MAPK reduces the levels of MMP3 in order to activate OPN, which is located on the cell membrane. This activation then initiates signaling pathways that promote osteoblastic cellular responses, leading to the production of new bone. Treatment of dexamethasone-induced zebrafish larvae with Z-VA resulted in increased expression of OPN and MAPK in the regenerated scales while decreasing the MMP3 levels [40].

In contrast, in zebrafish larvae treated with dexamethasone, increased levels of MMP3 caused the deactivation of OPN protein by breaking it down through the MAPK signaling mechanism. This resulted in reduced levels of MAPK in the regrown scales. Therefore, Z-VA shows potential as an anti-osteoporosis agent by correcting irregularities in bone resorption and the generation of new bone through the activation of the MMP3–OPN–MAPK signaling pathway [37].

### 3.9 Z-VA promotes bone fracture healing zebrafish fin fracture model

**Figure 10:**
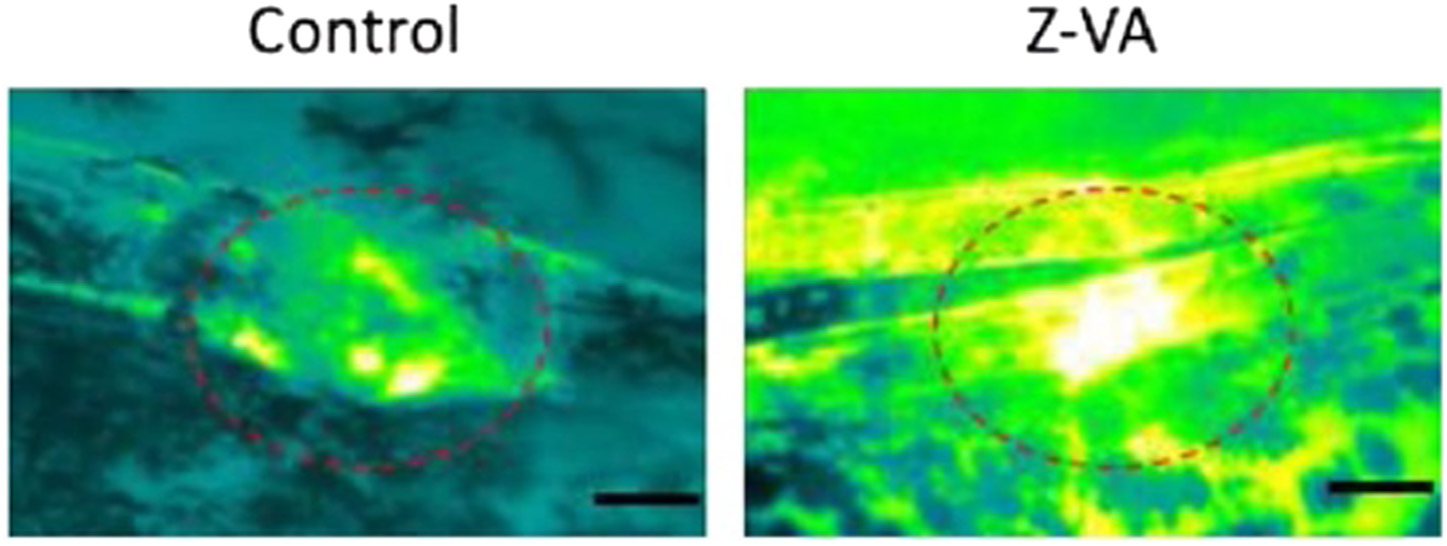
Z-VA promotes bone healing in the zebrafish fin fracture model. Zebrafish with induced fin fractures were treated with Z-VA for up to 14 days, and the formation of fin bone callus was observed. The scale bar indicates 500 µm.

The ability of Z-VA to promote bone formation was evaluated using a zebrafish fin fracture model in a live animal. The fins that had been severed and treated with Z-VA were analyzed at various time intervals. This analysis took into account that the mineralization of bone callus is mainly driven by the deposition of calcium by osteoblasts. Photographs of zebrafish fins that had suffered crush injuries, depicting their breakage and recovery on day 4 and day 18, were analyzed (Figure 10). The results clearly and definitively show that Z-VA has the ability to induce bone growth, resulting in complete bone regeneration by the third week. The observed advancement in fin remodeling highlights the ability of chito-oligosaccharides to generate bone, which has a major impact on the creation of skeletal callus in the severed fin region. Calcein staining assays were conducted to confirm the presence of mineralization in the regenerated fin. The results of these tests indicate that Z-VA has osteo-inductive qualities that stimulate the activation of cells responsible for bone formation, leading to increased calcium deposition at the place where the fin is regenerated. Osteoblasts play a crucial role in the formation of new bone and the preservation of the strength and structure of hard tissues. Our findings consistently demonstrate that Z-VA, with its cell adhesion factors, promotes the recruitment of osteoblasts to deposit calcium at the healed location. The existence of calcified nodules at the repaired fin bone location emphasizes the ability of chito-oligosaccharides to stimulate osteoblastic activity and enhance calcium absorption at the bone callus region, indicating their osteo-inductive action. To summarize, the crush injury model demonstrates the unique osteogenic capability of Z-VA in improving the integration of bone and promoting the regeneration of a well-developed fin callus. These findings align with previous studies on the healing of fin fractures, confirming the effectiveness of Z-VA in stimulating bone repair.

### 3.10 Effect of Z-VA on bone tissue parameters and serum biochemicals in a rat osteoporosis model

Our study examined the preventive effects and probable mechanisms of Z-VA in preventing osteoporosis induced by dexamethasone in rats. The details of the study may be found in Table 1. Body weight is a crucial determinant of general well-being and can directly reflect the effects of medications on physical processes. Remarkably, our study found that the administration of medications had no substantial impact on the body weight gain of rats. This indicates the dependability of our data and the lack of noteworthy adverse effects resulting from drug administration. BMC and BMD are important measures used to evaluate bone quality and are strongly linked to the severity of osteoporosis. The length, transverse diameter, and weight of the femur are directly indicative of bone quality [41]. Our findings indicate that the parameters in the dexamethasone group were lower compared to those in the control group, suggesting the successful establishment and administration of the osteoporosis model. Furthermore, alendronate, a well-established medication for treating osteoporosis, successfully restored the impact induced by dexamethasone, as demonstrated by Qaseem et al. [42]. This outcome confirms the effectiveness of our model and treatment. Similarly, Z-VA (10, 20, or 40 mg/kg), indicates a protective impact against dexamethasone-induced osteoporosis by enhancing the bone density. ALP is an enzyme that is released by osteoblasts and serves as a marker for bone formation [15]. Glucocorticoids, such as dexamethasone, have the ability to stimulate bone resorption and disrupt normal bone remodeling processes, ultimately resulting in the development of osteoporosis [43].

**Table 1:** Influence of Z-VA on the weight, length, transverse diameter, BMC, and BMD of the left femur in rats with osteoporosis

Group	Weight (g)	Length (mm)	Transverse diameter (mm)	BMC (g/cm)	BMD (g/cm^2^)
Control	0.69 ± 0.06	32.51 ± 1.05	3.44 ± 0.19	0.16 ± 0.05	0.28 ± 0.04
Dexamethasone	0.65 ± 0.05	32.12 ± 1.45	3.34 ± 0.24	0.13 ± 0.03	0.24 ± 0.05
Z-VA (10 mg/kg)	0.70 ± 0.07*	32.51 ± 1.71	3.49 ± 0.26*	0.16 ± 0.09	0.29 ± 0.14
Z-VA (25 mg/kg)	0.77 ± 0.06**	32.89 ± 1.38	3.89 ± 0.23**	0.23 ± 0.04**	0.36 ± 0.06**
Z-VA (50 mg/kg)	0.74 ± 0.05*	32.56 ± 1.44	3.77 ± 0.27*	0.22 ± 0.04*	0.33 ± 0.05*
Alendronate	0.75 ± 0.06*	32.57 ± 1.67	3.75 ± 0.22*	0.21 ± 0.06*	0.34 ± 0.05*

*indicates statistical significance compared to control (*p* < 0.05) and **indicates statistical significance compared to control, Z-VA (10 mg/kg), Z-VA (50 mg/kg), or alendronate (*p* < 0.05).

The results (Table 2) of our study showed that Z-VA (at doses of 10, 20, or 40 mg/kg) effectively reversed the dexamethasone-induced effect. Dexamethasone treatment decreased the ALP, interleukin-6 (IL-6), tumor necrosis factor-alpha (TNF-α), serum calcium (Ca^2+^), and serum inorganic phosphorus (IP) levels. Z-VA (at doses of 10, 20, or 40 mg/kg) or alendronate treatment reversed the dexamethasone-induced effect by increasing ALP, IL-6, TNF-α, Ca^2+^, and IP levels. This suggests that Z-VA has a protective effect against dexamethasone-induced osteoporosis by regulating the bone metabolism. IL-6 and TNF-α are cytokines that promote inflammation and have the ability to increase the breakdown of bone tissues while suppressing the production of new bone [44]. In summary, our research indicates that Z-VA has a beneficial impact on preventing dexamethasone-induced osteoporosis in rats. This is achieved by enhancing the bone density and modulating the bone metabolism. These findings emphasize the potential therapeutic benefits of Z-VA in preventing and treating osteoporosis. However, additional studies are necessary to understand its mechanisms of action and improve treatment plans.

**Table 2:** Impact of Z-VA on ALP, TNF-α, IL-6, serum Ca^2+^, and IP levels or activity in rats with osteoporosis

Group	ALP (U/L)	TNF-α (ng/L)	IL-6(ng/L)	Ca^2+^ (mmol/L)	IP (mmol/L)
Control	255 ± 19	85 ± 6.21	100 ± 4.2	2.70 ± 0.11	1.23 ± 0.22
Dexamethasone	245 ± 21	79 ± 5.15	81 ± 3.9	2.35 ± 0.21	1.17 ± 0.16
Z-VA (10 mg/kg)	256 ± 27*	87 ± 4.31*	96 ± 6.1*	2.71 ± 0.22*	1.27 ± 0.26*
Z-VA (25 mg/kg)	289 ± 36**	98 ± 6.61**	109 ± 8.4**	2.99 ± 0.25**	1.35 ± 0.19*
Z-VA (50 mg/kg)	267 ± 15*	86 ± 4.11*	100 ± 4.9*	2.76 ± 0.21*	1.26 ± 0.29*
Alendronate	280 ± 26*	97 ± 5.07*	103 ± 5.5*	2.71 ± 0.24*	1.27 ± 0.11*

*indicates statistical significance compared to control (*p* < 0.05) and **indicates statistical significance compared to control, Z-VA (10 mg/kg), Z-VA (50 mg/kg), or alendronate (*p* < 0.05).

## 4 Conclusions

In conclusion, the findings of this study show that Z-VA has potential as a new drug for both preventing and treating osteoporosis. Numerous *in vitro* and *in vivo* experiments performed with Z-VA have revealed several important osteogenic functions and anti-osteoporotic effects of this substance. Initially, mouse MSCs are not affected by Z-VA, which allows the compound to be biocompatible and safe for possible therapeutic uses. Additionally, when differentiated into osteoblasts, Z-VA significantly increases the ALP activity and calcium deposition compared with controls; this suggests the capacity of the substance to stimulate mineralization and promote differentiation into osteoblasts. Furthermore, bone formation in the dexamethasone-induced zebrafish model treated with Z-VA and in rat models of osteoporosis showed enhanced bone growth, density, and mineralization, as indicated by increased ALP activity in differentiated osteoblasts compared to controls. In addition to these findings, Z-VA also alters the expression pattern of key markers of bone formation such as Runx2, type 1 collagen, osteocalcin (BGLAP), osteonectin (SPARC), MAPK pathway (MAPK), and OPN (SPP1). It was also used on zebrafish fin fracture models, showing that it helped regrow broken bones and fin callus without causing any toxicity, which means it is very effective for healing bones. Additionally, in zebrafish fin fracture models, Z-VA promoted new bone formation and fin callus regeneration without inducing toxicity, highlighting its efficacy in bone healing and regeneration. Furthermore, in a rat model of osteoporosis, Z-VA exhibited protective effects against dexamethasone-induced bone loss by promoting bone mass and regulating bone metabolism. These effects were evidenced by improvements in bone tissue parameters, serum biochemical markers, and inflammatory cytokine levels. Overall, the comprehensive evaluation of Z-VA’s osteogenic potential across various experimental models underscores its promising role in osteoporosis management and bone tissue regeneration. Further research to elucidate its mechanisms of action and optimize therapeutic dosages is warranted to harness the full therapeutic potential of Z-VA in clinical settings.

## Supplementary Material

Supplementary Table
