# Supplementary material for: Zinc valproic acid complex promotes osteoblast differentiation and exhibits anti-osteoporotic potential
Source: Open Life Sci. 2025 May 12;20(1):20251090. doi: 10.1515/biol-2025-1090 (PMC12086624; doi:10.1515/biol-2025-1090)
Supplement: Supplementary Table [file biol-2025-1090-sm.pdf]

Supplementary material

Table S1: Primer sequences used for real time RT-PCR analysis

Gene		5′→3′ sequence
Runx2	Forward	CAGTTCCCAAGCATTTCATC
	Reverse	TCAATATGGTCGCCAAACAG
Type-1 collagen	Forward	TAACCCCCTCCCCAGCCACAAA
	Reverse	TTCCTCTTGGCCGTGCGTCA
GAPDH	Forward	TTGATGTCATCATACTTGGCAGGT
	Reverse	CAG TCAAGGCTGAGAATGGGA
mir-143	Forward	CAGTGCTGCATCTCTGGTCA
	Reverse	TGCAGAACAACTTCTCTCTTCCT
U6	Forward	CTCGCTTCGGCAGCACA
	Reverse	AACGCTTCACGAATTTGCGT
runx2a masns-	Forward	CTCCCGCTTTAGGACTTCGA
isoform	Reverse	GGAGTCACCGAGCTGAAAAGACT
Collagen 1α2	Forward	GGAAACCTGAAGAAGGCTGTGT
	Reverse	TGAAAGTGAAGCGGCTGTTG
Osteocalcin	Forward	TGGCCTCTATCATCATGAGACAGA
	Reverse	CTCTCGAGCTGAAATGGAGTCA
Osteopontin	Forward	CGCTCAGCAAGCAGTTCAGA
	Reverse	AGAATAGGAGGTGGCCGTTGA
β-actin	Forward	CAACAGGGAAAAGATGACACAGAT
	Reverse	CAGCCTGGATGGCAACGT
